# Selective Thermal Transformation of Automotive Shredder Residues into High-Value Nano Silicon Carbide

**DOI:** 10.3390/nano11112781

**Published:** 2021-10-20

**Authors:** Sepideh Hemati, Rumana Hossain, Veena Sahajwalla

**Affiliations:** Centre for Sustainable Materials Research and Technology, SMaRT@UNSW, School of Materials Science and Engineering, UNSW, Sydney, NSW 2052, Australia; s.hemati@unsw.edu.au (S.H.); veena@unsw.edu.au (V.S.)

**Keywords:** automated shredder residue, end-of-life-vehicles, nano silicon carbide, windshield glass, waste recycling

## Abstract

Automotive waste represents both a global waste challenge and the loss of valuable embedded resources. This study provides a sustainable solution to utilise the mixed plastics of automotive waste residue (ASR) as a resource that will curtail the landfilling of hazardous waste and its adverse consequences to the environment. In this research, the selective thermal transformation has been utilised to produce nano silicon carbide (SiC) using mixed plastics and glass from automotive waste as raw materials. The composition and formation mechanisms of SiC nanoparticles have been investigated by X-ray diffraction (XRD), X-ray-Photoelectron Spectroscopy (XPS) and Transmission Electron Microscopy (TEM). The as synthesised SiC nanoparticles at 1500 °C has uniform spherical shapes with the diameters of the fixed edges of about 50–100 nm with a porous structure. This facile way of synthesising SiC nanomaterials would lay the foundations for transforming complex wastes into value-added, high-performing materials, delivering significant economic and environmental benefits.

## 1. Introduction

The management of waste material and the limited resources within our planet is one of the most critical challenges that engineers, and scientists are facing to solve. Australia produced about 67 million tonnes of waste in 2016–2017 [1]. The biggest resource-consuming and waste producer industry among different industry sectors is the automotive industry [2]. The volume of waste material generated from end-of-life-vehicle (ELVs) is indeed enormous that requires to be appropriately managed. The waste management process of end-of-life-vehicle (ELVs) can be categorised in three broad stages: (I) depollution, (II) dismantling and (III) shredding [3]. Around 70–75% of total shredded output contains ferrous fraction and nonferrous metals, while the 20% to 25% of remaining from shredded production is named the automotive shredder residue (ASR) [4]. Generally, ASR is found after the separation of metals from the shredded materials which usually contains 19–31% plastic, nearly 20% rubber, 10–42% textile and fibre materials, 2–5% wood residues, and 5.2% glass [5,6]. Previous research showed that ASR can be an excellent source of carbon because of the presence of substantial amount of wood and plastic [7].

Sorting and recovering the value-added resources from mixed waste plastics of ASR include two main categories; the mechanical classification of waste that is recyclable and the thermal treatment of waste for recovering value-added materials or energy-producing fuels [3]. Gasification technologies are increasingly considered the ideal choice for the thermal processing of waste materials because these renewable processes decrease the need for fossil fuels and reduce greenhouse gas emissions [8,9]. Recently, the thermal process is receiving attention because of its flexibility to produce different proportions in a combination of solid, liquid, and gaseous products with a wide range of variations in the operating factors such as temperature rate, time, thermal treatment atmosphere, etc. and providing an opportunity for transforming low-energy-density materials into high-energy-density biofuels [10,11]. Numerous studies have been conducted on thermal treatment of waste from the automotive such as tyre, synthetic polymers, acrylic fibres, mixed plastics to produce oil, gas, and carbonaceous product [12,13,14,15,16,17]. Some recent studies showed that mixed glass from the auto waste can be a very good source of silica [18]. Still, there is a lack of research on the commingled glasses found from the automotive waste. In addition, there are a few studies on the thermal treatment of mixed waste plastics from auto waste for the nanomaterials. Still, the thermal energy recovery from waste plastics from ASR has not been commercially and technically proven and requires further investigations. Besides, most previous research has studied the thermal transformation of ASR at moderate temperatures between 450 °C and 800 °C [19,20,21,22,23,24,25,26,27]. Consequently, there is a lack of understanding associated with the use of high temperatures and the rapid heating of carbon-bearing wastes containing oxides and their transformations under such extreme condition—conditions that usefully minimise the generation of toxic gases. This research aimed to find a facile solution to transform hazardous mixed plastic and glass waste into valuable products and resources which could be the feedstock to other manufacturing industries. Silicon carbide is an advanced ceramic compound with excellent thermomechanical, high temperature/corrosive resistance, chemical stability and electrical properties [28].

Nanostructured SiC materials not only possess the excellent properties of its bulk but also exhibits the unique properties of nanomaterials, for example, small-sized effect, surface and boundary effect, quantum size effect [29,30]. Hence the nanostructured SiC materials can be applied in the field of nanoscale electro-devices, nanosensors, biological labels, and light-emitting devices [31,32,33,34,35,36]. Several efforts have been devoted to preparing various types of nanostructured SiC. The most common methods are sol-gel [37,38,39], chemical vapor deposition [40,41], plasma [42,43], microwave heating [44], etc. Most of the abovementioned methods for preparing nanostructured SiC used expensive raw materials. Recently, the use of waste materials such as agricultural waste [45], plastic waste [46,47], biomass gasification [48] as a carbon source, and glass waste and e-waste as silicon source in the production of nanostructured SiC has been studied [49,50,51]. Researchers have utilised the waste tyres as a carbon source to produce nano SiC [51,52]. However, producing nano SiC by utilising waste glasses and mixed plastics of automotive waste as the silicon and carbon sources without using any excessive raw materials is the main objective of this study. It represents a sustainable thermal approach to utilise the automotive waste to fabricate nanostructured SiC, simultaneously reducing pressure on virgin resources, aiding waste management, and producing value-added high performing materials.

## 2. Material and Methods

### 2.1. Raw Materials

**Figure 1:**
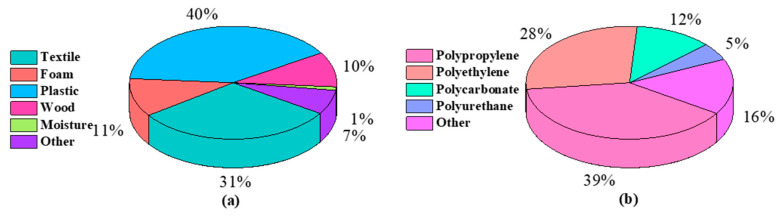
(**a**) ASR compositions, and (**b**) plastic composition of ASR [26,53,54,55].

In this study, because of the accessibility and availability to the local manufactures, the vehicles windshield glass waste and the mixed plastics of ASR were collected from the local steel manufacturer in Australia. Table 1 represents oxides in the windshield glass powder; the oxides less than 0.1 wt% are ignored. Silica is the dominant oxide of automotive glass with a mass fraction of 71.25% and other major oxides in automotive glass are Na_2_O, CaO and MgO, with mass fractions of 15.50%, 8.30%, 3.73%, respectively. The XRF result represents that the windshield glass is a suitable source of silica. As listed in Figure 1: (a) ASR compositions, and (b) plastic composition of ASR [26,53,54,55], ASR is a complex mixture of a wide range of materials such as textiles, wood, foam, and different plastic types [24,51,52,53]. According to Figure 1, ASR comprises 40% plastic, and the main plastic-type in the ASR is polypropylene 39%. The percentages of carbon (C), hydrogen (H), nitrogen (N), sulphur (S) and oxygen (O) of raw ASR are measured via CNHSO elemental analysis. As summarised in Table 2, CHNS analysis of ASR, the carbon percentage in ASR is 25.74%, which indicates that the ASR is a potential source of carbon.

### 2.2. Experimental

The waste glass and the mixed plastic of ASR were grounded separately by Pulveriser Ring mills metal screen in size range of 0.75–1 mm for 2–3 min and 10 min, respectively. Then the waste glass powder and ASR plastics powder were mixed with 30–70% weight ratios to make pellets using the Carver hot press (Model 2697, Menomonee Falls, WI, USA) at 150 °C with a uniaxial pressure (200 bar). Then pellets were dried in the oven for more than 12 h at 60~90 °C to remove any moisture content. Next, the pellets were placed in a horizontal tube furnace (Model HTF 7060, Ceramic Engineering, Furnace Manufacturers Sydney, Australia) under high purity argon (>99%) gas supply at 0.8 L min^−1^ using a mass flow controller. The heating chamber temperature was 700 °C to 1500 °C, and the holding time was 2 h. The as-synthesized products were collected and then purified by diluted HF solution. The purified products were further washed by deionized water to neutral, and the pure products were obtained after drying in an oven. The samples were grounded by mortar and pestle for further analysis.

Thermo-gravimetric analysis (TGA, STA-8000, PerkinElmer, Groningen, Netherlands) was conducted from room temperature to 850 °C at 20 °C min^−1^ in a nitrogen-enriched atmosphere with a purified rate of 20 mL min^−1^ to characterise the thermal transformation behaviour of raw materials under the influence of heating. The primary chemical group and gas identification of thermal decomposition of mixed powder ASR plastics and glass were analysed by Fourier transform infrared spectroscopy (FTIR, Spotlight 400 FTIR, PerkinElmer, Llantrisant, UK). Chemical composition and major oxides of raw materials were studied via X-ray fluorescence spectroscope analyser (XRF, instrument AXIOS, WD-XRF with Rh end-window tube, PANalytical, EA Almelo, Netherlands), “SUPERQ” software, and WROXI 1222 program was used for substantial elements which expressed as major oxides. X-ray-photoelectron spectroscopy (XPS, instrument ESCALAB250Xi, Thermo Scientific, Loughborough, UK) examined the chemical state of the surface of raw and heat-treated materials. X-ray source was monochromatic Al K-alpha with energy 1486.68 eV, the power of 120 W (13.8 kV, 8.7 mA), and binding energy reference C1s 284.5 eV for graphite. To characterise and obtain information about the phase identification and crystallinity of raw materials and synthesized SiC X-ray diffraction (XRD, Empyrean II, PANalytical, Eindhoven, The Netherlands) analysis was performed with Co Anode, K-alpha 1.78901 Å at 40 mA and 45 kV, with XRD ranged from 10° 2θ to 110° 2θ. The XRD results were further analysed by high score plus software (version 5.1, 2021, Malvern PANalytical, Eindhoven, Netherlands). Specific surface areas and pore size distributions were detected via the Brunauer–Emmett–Teller (BET, instrument type 3020, Nitrogen at temperature 77 K, Norcross, GA, USA) and BJH models. Transmission electron microscopy (TEM, Philips CM 200, FEI Company, Eindhoven, Netherlands) was used in this study to identify morphology, high-resolution transmission electron microscopy (HRTEM)and selected area electron diffraction (SAED) to detect lattice structure and diffraction pattern.

## 3. Results and Discussion

ASR is a complicated solid waste as it is an incredibly diverse mixture of residual metals, polymers (plastics, rubber, textile, foam) and inorganic fillers (ash and glass). Polypropylene (PP), polyethylene (PE), polyvinyl chloride (PVC), acrylonitrile butadiene styrene (ABS) and polyamide (PA) are polymer materials that could be the main parts of ASR [56,57]. Other trace components which could be seen in ASR are small glass pieces or metals. Thus, to obtain a characterisation such as thermal transformation behaviour and gas evaluation, TGA, FTIR, and find composition XPS, XRD analyses were conducted, respectively.

### 3.1. Characterisation of Raw Materials

**Figure 2:**
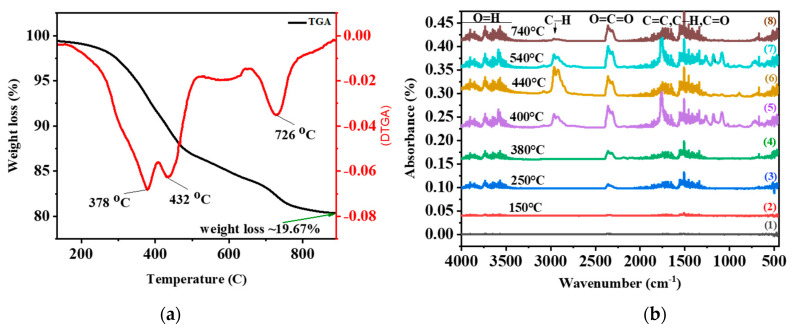
(**a**) TGA and DTGA curves of mixed 50–50% the ASR plastics and glass, (**b**) FTIR analysis of mixed powder of the mixed plastics of ASR and glass at different periods (**1**) 3 min, (**2**) 5 min, (**3**) 10 min, (**4**) 15 min, (**5**) 22 min, (**6**) 25 min, (**7**) 30 min and (**8**) 40 min.

Thermo-gravimetric analyses (TGA) and differential thermo-gravimetric analysis (DTGA) of the mixed plastics of ASR and glass are illustrated in Figure 2a. TGA analysis was carried out by heating mixed ASR plastics and glass from ambient temperature to 850 °C at 20 °C min^−1^ in a nitrogen atmosphere with a purify rate of 20 mL min^−1^. As depicted Figure 2a, the thermal weight loss of mixed ASR plastics and glass was started from 160 °C (*T*_onset_) and ended at 800 °C (*T*_endset_). The maximum thermal weight loss (*T*_max_) for combined ASR and glass occurred around 378 °C. Moreover, Figure 2a displays the multi-staged reduction in the TGA curve as the high mass fraction results. The plastics in the ASR cause the first two degradation step in the TGA curve at 378 °C and 430 °C, respectively, and the last degradation step is due to the glass transition at 726 °C.

As shown in Figure 2b, FTIR analysis of the mixed powder ASR plastics and glass thermally degraded at different temperatures and different time periods was conducted. Plastics of ASR released the hydrocarbon gases visible up to a specific temperature from 400 °C to 540 °C. Peaks between 2300 cm^−1^ and 2360 cm^−1^ could be assigned to CO_2,_ which shows CO_2_ release increased as temperature increased until 540 °C after this temperature at 740 °C still there is CO_2_ peak but intensity decrease [58]. As shown in Figure 2b, the absorbance bands were different before 400 °C and after 540 °C, but the location of the absorbance bands from 400 °C to 540 °C was almost the same; however, the intensities were different. This could conclude that the heat temperature caused each product’s yield, and the main categories of volatile products were not affected by temperature [58]. The largest signals are shown in 440 °C for 25 min graph (6). Figure 2b indicates wavenumber ranges of absorption peaks vs. the percentage absorption at different time–temperature profiles. The IR absorption bands around 3500–3900 cm^−1^ could be assigned to hydroxyl group O=H and which are symmetric and asymmetric stretch [59]. The band observed in Figure 2b in the graph 5 and 7 at around 2914 cm^−1^ is attributed to –CH_2_– symmetric stretch, and the band observed about 2960 cm^−1^ is assigned to asymmetric stretch isopropyl functional group (–CH_3_) in polypropylene [53]. The IR bands at 1536 cm^−1^, 1455 cm^−1^ and 1376 cm^−1^ are attributed to polyethylene single bond –CH_3_ and single bond –CH_2_ [60]. Figure 2b indicates a carbonyl (O=C=O) stretch with a dipole moment and two signals at 2300 cm^−1^ and 2360 cm^−1,^ implying symmetric and asymmetric stretching. The band at 2300 cm^−1^ is a symmetric stretch with a strong absorbance in the double bond region. The asymmetric stretch takes more energy; therefore, the higher signal at 2360 cm^−1^ is an asymmetric stretch. As shown in Figure 2b, the bands around 1260–2000 cm^−1^ are attributed to C=C, C–H, C=O. Figure 2b shows that the band in graphs f and g around 1750 cm^−1^ is related to C=O stretching mode of the amide functional group in polyurethane. Moreover, the bands around at 1255 cm^−1^ and 1020 cm^−1^ are attributed to the C–C–N bending and C–O–C anti-symmetrical stretch in polyurethane [53]. As shown in Figure 2b, the IR bands in graphs 5 and 7 about 740 cm^−1^ are allocated to the CH out of plane deformation of ortho-disubstituted benzene, and 1495 cm^−1^ are assigned to the stretching mode of the benzene ring in polycarbonate [53]. Therefore, the FTIR spectrum confirmed that ASR contained polypropylene (PP), polyethylene (PE), polyurethane (PU) and polycarbonate (PC) polymers.

**Figure 3:**
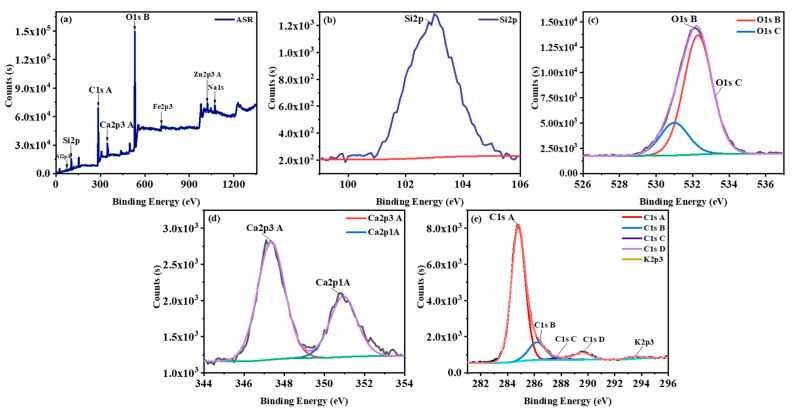
(**a**) XPS survey scan of the mixed plastics of ASR and typical high-resolution XPS spectra of (**b**) Si2p, (**c**) O1s, (**d**) Ca2p, (**e**) C1s.

XPS analysis was conducted to explore the chemical state of the elemental surface of the mixed plastics of ASR. As shown in Figure 3 C1s A and O1s C are the most substantial peaks, and other peaks of Si2p, Ca2p implies on there are inorganic fillers, for example, fibreglass, calcium carbonate and other mineral oxides [60,61]. Moreover, carbon, oxygen and chlorine demonstrated in Figure 3a could originate from polymer materials such as PP, PE, ABS, PA, PU and PVC. Additionally, Figure 3a reveals aluminum and iron peaks which could come from the metal element in ASR. As summarised in Figure 3a peaks around 103 eV, 285 eV, 347 eV, 532 eV and 1072 eV with concentrations of the formed atom, 8.64%, 36.51%, 2.64%, 30.58% and 1.36% correspond to Si2p, C1s A, Ca2p^3^ A, O1s B and Na1s, respectively. Figure 3d depicted the Ca2p^3^ A peak which, according to the NIST database, the peak around 346.5–347.0 eV are assigned to CaCO_3_. Based on Choi, M. et al., [62] the peak around 102 eV confirmed the silicon atom is the electrovalence. According to Farzana, R. et al., [49] the most substantial peak was for O1s with binding energy 532 eV; this could be as a result of the presence of oxygen from the exposure of the materials to the atmosphere.

**Figure 4:**
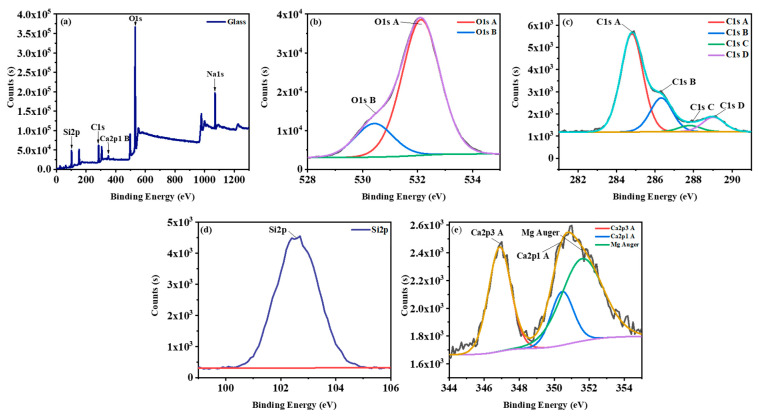
(**a**) XPS survey scan of glass from automotive waste and typical high-resolution XPS spectra of (**b**) O1s, (**c**) C1s, (**d**) Si2p, (**e**) Ca2p.

XPS analysis was conducted to obtain information about the chemical compounds’ state on the surface of the glass from automotive waste. As summarised in Figure 4a, the binding energy of Na1s, O1s, Ca2p1 B, C1s, Si2p peaks are around, 1071 eV, 532 eV, 351 eV, 285 eV, 102 eV, respectively. The C1s peak is called adventitious carbon caused by the adsorption of hydrocarbon impurities which does not affect interpreting the results [63]. C1S is related to the sp^2^ carbon bonding (sp^2^C) at 284.3 ± 0.15 eV, sp^3^ carbon bonding (sp^3^C) at 285.0 ± 0.1 eV, C–O bonding at 286.2 ± 0.1 eV and C=O bonding at 287.9 ± 0.1 eV [64,65]. O1s is the highest peak. The high-resolution XPS spectrum of O1s shows two peaks around 532 eV and 530 eV with an atomic percentage of 41.94% and 10.37%, respectively. According to Liste, S. et al. [66], 532 eV refers to the Si–O–Si vibration (bridging oxygen groups, BO) and the value of 530 eV is associated with the nonbridging silicon-oxygen groups (NBO).

**Figure 5:**
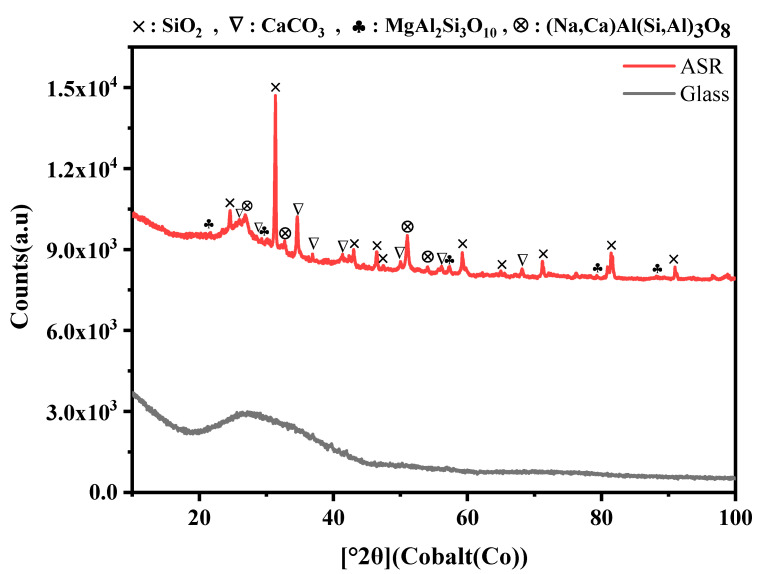
XRD spectrums of the mixed plastics of ASR and glass.

Figure 5 demonstrates the XRD pattern of the mixed plastics of ASR and glass. According to Figure 5, the XRD pattern of glass shows a broad peak which indicates glass was used in this study has an amorphous nature. Additionally, based on the information from An, Z. et al., [67] a broadened dispersion peak at 22° with no sharp diffraction peak corresponds to the occurrence of amorphous materials. Moreover, the XRD pattern reveals the lack of any ordered crystalline structure [68]. Figure 5 shows the XRD pattern of the mixed waste plastics of ASR, which identified the main crystalline phases. The ASR graph shows that the mixed plastics of ASR mainly contains SiO_2_, CaCO_3_, MgAl_2_Si_3_O_10_, and (Na,Ca)Al(Si,Al)_3_O_8_. The strongest peak in the XRD pattern of ASR belongs to Si_2_O. The quantitative XRD analysis suggests that the volume percentage of the MgAl_2_Si_3_O_10_ phase are less than 1% ASR plastics.

### 3.2. Thermal Transformation of the Waste Glass and Mixed Plastics of ASR

**Figure 6:**
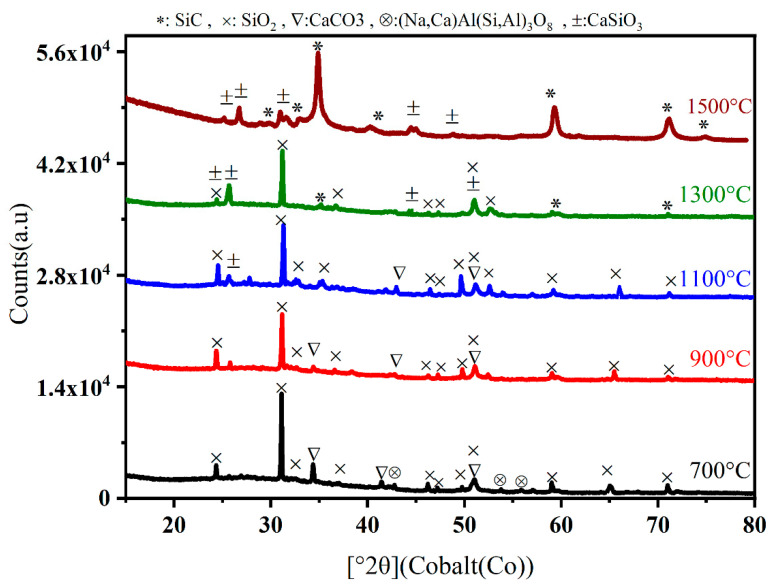
XRD spectrums of heat-treated mixed ASR plastics and glass at 700 °C to 1500 °C for 2 h.

In this paper, glass from the windshield of cars was used as a Si source and ASR plastics as a source of C to synthesise high-value materials. X-ray diffraction spectra indicate that SiC did not form up to the temperature of 1100 °C. Only peaks of SiO_2_ were identified, which suggests that the thermal energy was not sufficient to trigger the reaction between the SiO_2_ of the glass and C of the ASR plastics. Figure 6 demonstrates that the formation of SiC has been started at 1300 °C, and by increasing the temperature to 1500 °C, the production of SiC was also increased. Therefore, the results suggest that the reaction between Si and C happens at a higher temperature, confirmed by the other researchers [51]. SiC peaks at 1500 °C are stronger than 1300 °C. At 1500 °C, there are no SiO_2_ peaks observed; just a minor peak of SiO_x_C_y_ could be seen, which suggests an almost complete reaction between the SiO_2_ of glass and C of ASR plastics for the total transformation into SiC. The highest peak for SiC is around 35.69°. The SiC in both 1300 °C and 1500 °C has a cubic crystal system.

**Figure 7:**
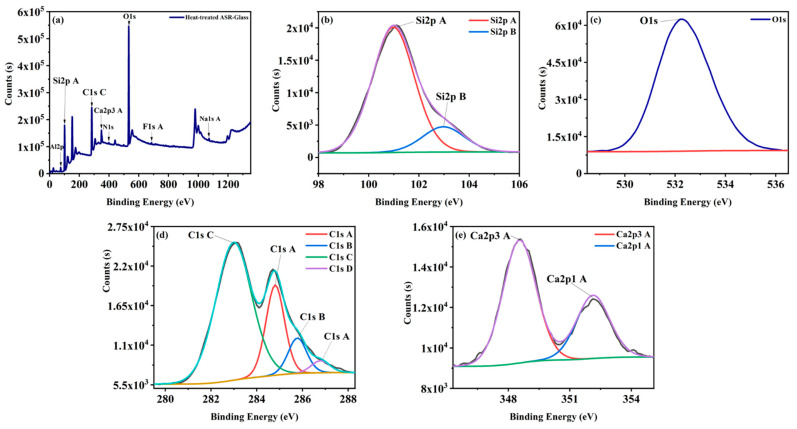
(**a**) XPS survey scan of heat-treated mixed ASR plastics and glass at 1500 °C for 2 h and typical high-resolution XPS spectra of (**b**) Si2p, (**c**) O1s, (**d**) C1s, (**e**) Ca2p.

X-ray photoelectron spectroscopy (XPS) was used to study the chemical phenomenon occurring at surfaces of materials. XPS analysis of mixed ASR plastics and glass at 1500 °C was conducted. The results of samples at 1500 °C illustrates the reduction of SiO_2_ with carbon to produce SiC. Additionally, Figure 7a and Table 3 depicts the highest peaks belonging to O1s with binding energy 532 eV with concentrations of the formed atom was 28.86%, which could result from contamination caused by exposure to oxygen in the atmosphere. C1s C, Si2p A, Ca2p^3^ A have high peaks around 283 eV, 101 eV, 347.57 eV with concentrations of the formed atom 22%, 29.96%, 1.84%, respectively. As shown in Table 3 and Figure 7b, Si2p has two peaks around 101 eV, 102.5 eV allocated to SiC and SiO_2_ [69,70,71]. High-resolution XPS spectrum of C1s indicates that the highest peak C1s C (283 eV) associated with high-coordination C–Si, and C1s A (284.8 eV) related to C–C as reported by McKenna, J. et al. [72]. As stated by Lee, K. H. et al. [73] Si2p with the binding energy of 101 eV–102 eV confirms the formation of the Si–C bond; whereas the C1s spectrum with the binding energy of 282 eV–284 eV demonstrates the development of Si–C bond. Moreover, according to Aitana et al. (2018) the peaks of Si2p and C1s spectra with the binding energy of 101 eV and 283 eV associated with the unoxidised Si and C atoms of the SiC [71,74]. As discussed by Rajarao, R. et al. [75] O1s with binding energy, 532 eV is related to Si–O–C bond in silicon oxycarbide. Subsequently, the XPS results confirm the formation of SiC.

**Figure 8:**
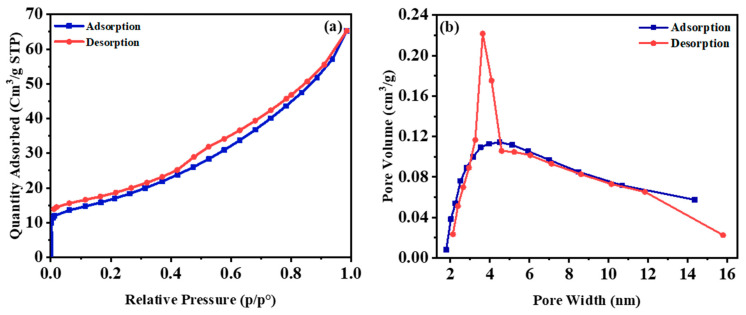
(**a**) Nitrogen adsorption–desorption isotherms of SiC nanoparticles, and (**b**) Pore size distribution (BJH) of SiC nanoparticles obtained at 1500 °C for 2 h.

Figure 8a plots the N_2_ adsorption–desorption isotherms of SiC representing the type-IV shape; this type of curves has the characteristic of mesoporous materials [90,91]. As depicted in Figure 8a, when the relative pressure is in the range of 0.5 p/p° to 0.9 p/p°, the adsorption and desorption isotherms are abruptly changed, which is one of the prominent aspects of mesoporous materials [92]. Moreover, the adsorption–desorption isotherms still rise above the relative pressure of 0.9 p/p°, which suggested that the sample has some macroporous [93]. According to BET analysis, the surface area is 58.8203 ± 0.2508 m^2^/g. Figure 8b, demonstrates that the sample’s pore width is between 2 and 16 nm. The maximum pore width is about 4.50 nm, indicating that the sample is mesopores.

**Figure 9:**
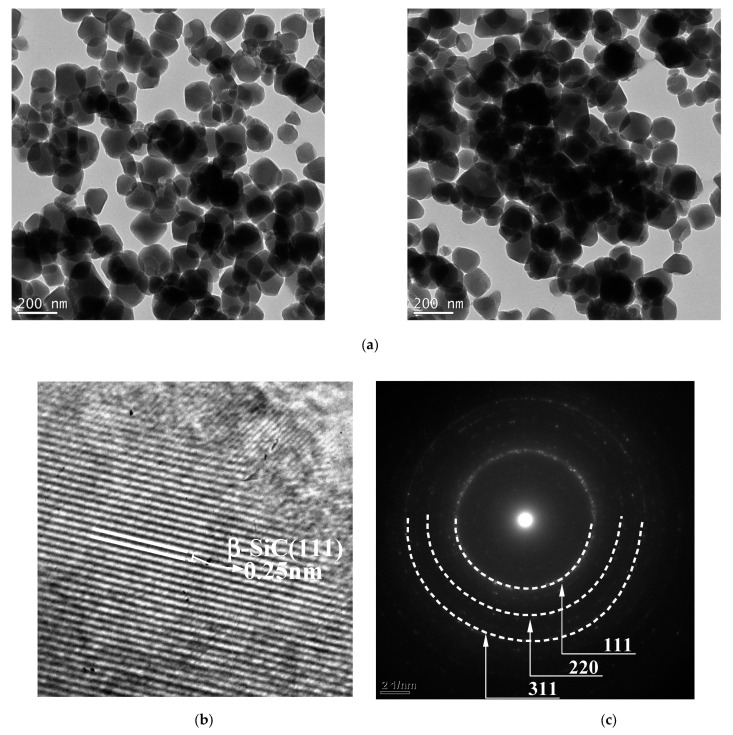
(**a**) A general low-resolution TEM image, (**b**) a representative high-resolution, (**c**) a corresponding selected area electron diffraction pattern of SiC nanoparticles obtained at 1500 °C for 2 h.

The structure and morphology of SiC nanoparticles obtained at 1500 °C for 2 h were characterised by using transmission electron microscopy (TEM). Figure 9a shows the low-resolution transmission electron microscopy (LRTEM) image of as synthesised SiC. As depicted in Figure 9a SiC are quasi-spherical particles with the length of the fixed edges of about 50–100 nm with a porous structure. These SiC particles are stacked together to form secondary particles with irregular pores. Figure 9b represents high-resolution transmission electron microscopy (HRTEM). It indicates SiC is highly crystallised, which means that nanoparticles are composed of many small single-crystalline SiC nanoparticles with different orientations. As presented by Wang et al. [94], the high crystallinity of SiC could be recognised by the regularly arranged lattice. XRD patterns of SiC nanospheres confirm that there are three perfect diffraction peaks at 35.69 °, 60.11 ° and 71.78 ° correspond to rings (111), (220) and (311) planes of cubic SiC (Reference code: 00-029-1129). HRTEM image demonstrates sharp lattice separation of (111) planes with d = 0.25 nm links to the cubic phase of SiC crystals. The peaks in Figure 9c illustrates the selected area electric diffraction pattern (SAED) of a single spherical nanoparticle. As depicted in Figure 9c the SAED patterns reveal that nanoparticle is polycrystalline SiC and each diffraction ring could be indicated to the (111), (220), (311) planes of the cubic SiC, respectively.

### 3.3. Formation Mechanism of the SiC Nanospheres

At a high temperature, thermal treatment isolated useful carbon from ASR plastics materials to produce carbon products and silica layers. The reaction mechanism of carbothermic reduction is explained from equation 1 to 6. The carbothermic reduction of SiO_2_ to synthesis SiC is one of the most efficient methods due to cheap raw materials and minimal reaction equipment [95,96,97,98]. The reaction temperature and the raw material have significant roles in synthesising the SiC nanosphere [99]. Based on the general reaction (6), SiO_2_ reacts with C and form SiC. However, the formation of SiC is a complicated and needs a series of solid–solid, solid–liquid, solid–gas and gas–gas reactions [100,101,102,103]. The synthesis of SiC is a multi-step reaction with the beginning of the reduction of SiO_2_ by C based reaction (1) in direct physical contact [104]. The mixed ASR plastics and glass were heated at 1500 °C for 2 h, during this time, SiO_2_ and C reacted, and SiO and CO were generated, as shown in reaction (1). Then, the reaction of CO with SiO_2_ results in the formation of SiO and CO_2_ (2). CO_2_ reacted with the surrounding C to form CO (5); this reaction boosted reaction (4) to grow SiC continuously [51,99]. Moreover, SiO and C could react together and produce SiC (3). As demonstrated in reaction (4), the SiO and CO reacted together and produced SiC. The reduction of SiO_2_ by the reaction between C and Si to synthesise SiC can be explained as follows [47,48,49].

SiO_2_(s,l) + C(s) → SiO(g) + CO(g), (1)

SiO_2_(s,l) + CO(g) → SiO(g) + CO_2_(g), (2)

SiO(g) + 2C(s) → SiC(s) + CO(g),(3)

SiO(g) + 2CO(g) → SiC(s) + CO_2_(g), (4)

CO_2_(g) + C(s) → 2CO(g). (5)

Based on An, Zibo et al., the general reaction is as follows [38].

SiO_2_(s,l) + 3C(s) → SiC(s) + 2CO(g) (6)

## 4. Conclusions

In this research, mixed glass and plastics from the automotive waste have been transformed thermally into the SiC nanoparticles. Thermal transformation from 700 °C to 1300 °C could not be converted the SiO_2_, and whatever trace of formation of SiC at 1300 °C confirmed by XRD indicated that slight partial transformation occurred at this stage. Hence the results show that at low temperature the chance of formation SiC is low. Accordingly, the results demonstrate that the carbothermic reduction has taken place at 1500 °C, and with the presence of Si and C in waste glass and mixed plastics of ASR, silicon carbide was synthesised. The characterisation of the sample obtained after the thermal transformation at 1500 °C confirms SiC nanoparticles with the diameter of about 50–100 nm. The BET surface area of the as synthesised material is 58.82 ± 0.25 m^2^/g with a pore diameter between 2 and 16 nm. Converting the problematic mixed waste plastics and glass waste into refined nanomaterials is promising for the sustainable solution of the automotive waste, which will otherwise be destined to be landfilling.

## Figures and Tables

**Table 1:** Major oxides in the windshield glass done by XRF.

Oxide	(wt%)
SiO_2_	71.25
Na_2_O	15.50
CaO	8.30
MgO	3.73
Al_2_O_3_	0.47
Fe_2_O_3_	0.46
K_2_O	0.13
TOTAL	99.84

**Table 2:** CHNSO analysis of ASR.

N (%)	C (%)	H (%)	S (%)	O (%)
0.72	25.74	3.002	0.258	11.66

**Table 3:** Typical photoelectron binding energies of heat-treated sample at 1500 °C for 2 h.

Name	Peak BE	Atomic%	Possible Compounds	Ref.
Si2p A	101	29.96	SiC	[70,71]
Si2P B	102.5	5.34	SiOC/CaSiO_3_	[69,76]
C1s C	283	22	SiC	[71,73,77]
C1s A	284.8	7.46	C	[72,78]
O1s	532	28.86	SiOC/MgO/Al_2_O_3_/CaSiO_3_	[75,76,79,80,81,82]
Ca2p^3^ A	347.54	1.84	CaSiO_3_	[76]
Al2p	75	3.88	Al_2_O_3_	[83,84,85]
Mg2p	51	0.10	MgO	[86]
N1s	399	0.13	pyridinic N	[87]
Na1s A	1073	0.23	NaCl/Cu	[88]
F1s A	686	0.2	Na_2_SiF_6_	[89]

## Data Availability

Data can be available upon request from the authors.
